# Review of 58 patients with necrotizing fasciitis in the Netherlands

**DOI:** 10.1186/s13017-016-0080-7

**Published:** 2016-05-27

**Authors:** Sander F. L. van Stigt, Janneke de Vries, Jilles B. Bijker, Roland M. H. G. Mollen, Edo J. Hekma, Susan M. Lemson, Edward C. T. H. Tan

**Affiliations:** Department of Surgery, Traumasurgery Radboud University Medical Center, Geert Grooteplein-Zuid 10, 6525 GA Nijmegen, The Netherlands; Department of Medical Microbiology, Radboud University Medical Center, Geert Grooteplein-Zuid 10, 6525 GA Nijmegen, The Netherlands; Department of Anesthesiology, Gelderse Vallei Hospital, Willy Brandtlaan 10, 6716 RP Ede, The Netherlands; Department of Surgery, Gelderse Vallei Hospital, Willy Brandtlaan 10, 6716 RP Ede, The Netherlands; Department of Surgery, Rijnstate Hospital, Wagnerlaan 55, 6815 AD Arnhem, The Netherlands; Department of Surgery, Slingeland Hospital, Kruisbergseweg 25, 7009 BL Doetinchem, The Netherlands

**Keywords:** Necrotizing fasciitis, Outcome, Soft tissue infections, LRINEC score, The Netherlands, ICU

## Abstract

**Background:**

Necrotizing fasciitis is a rare, life threatening soft tissue infection, primarily involving the fascia and subcutaneous tissue. In a large cohort of patients presenting with Necrotizing fasciitis in the Netherlands we analysed all available data to determine the causative pathogens and describe clinical management and outcome.

**Methods:**

We conducted a retrospective, multicentre cohort study of patients with a necrotizing fasciitis between January 2003 and December 2013 in an university medical hospital and three teaching hospitals in the Netherlands. We only included patients who stayed at the Intensive Care Unit for at least one day.

**Results:**

Fifty-eight patients were included. The mortality rate among those patients was 29.3 %. The central part of the body was affected in 28 patients (48.3 %) and in 21 patients (36.2 %) one of the extremities. Most common comorbidity was cardio vascular diseases in 39.7 %. Thirty-nine patients (67.2 %) were operated within 24 h after presentation. We found a type 1 necrotizing fasciitis in 35 patients (60.3 %) and a type 2 in 23 patients (39.7 %).

**Conclusions:**

Our study, which is the largest study in Europe, reaffirmed that Necrotizing fasciitis is a life threatening disease with a high mortality. Early diagnosis and adequate treatment are necessary to improve the clinical outcome. Clinical awareness off necrotizing fasciitis remains pivotal.

## Background

Necrotizing fasciitis (NF) is a part of the Necrotizing Soft Tissue Infections. It’s a rare, life threatening soft tissue infection, primarily involving the fascia and subcutaneous tissue. Although the symptoms were already described in the fifth century BC by Hippocrates [1, 2], even in modern medicine it still has a high mortality rate ranging from 6 to 76 % [1, 3–6]. The term necrotizing fasciitis was introduced by Wilson in 1952 [1, 7].

The rapidly progressive infection can affect any part of the body. The portal of entry usually is a minor injury of the affected site or a surgical wound. However, no definitive cause can be found in 20–50 % [8–10].

Medical conditions associated with necrotizing fasciitis are diabetes mellitus (31–44 %), obesity (28 %), smoking (27 %), alcoholabusus (17 %), cirrhosis (8–15 %), malignancy (3 %), corticosteroid therapy (3 %) and chronic renal failure (3 %) [11, 12].

The incidence of NF is low with 0.4 cases per 100.000 in the United Kingdom [13].

Clinical symptoms consist of local symptoms like erythema, swelling, changes in skin colouring, intense pain, bullae and sometimes subcutaneous emphysema and general symptoms such as fever, nausea, vomiting and malaise [2, 8, 11].

Necrotizing fasciitis can be classified in four clinical forms, depending on the causative organisms [2]. In Type 1 at least one anaerobic species is isolated with one or more facultative anaerobic streptococci (other than group A) and members of the Enterobacteriaceae (e.g., *E.coli, Enterobacter, Klebsiella, Proteus*) [14]. Type 2 is generally monomicrobial and caused by hemolytic streptococcus group A, sometimes with co-infection of *Staphylococcus aureus*. Most articles show type 1 is more common, with a relative incidence up to 75 % [8, 9]. Some studies also describe type 3, caused by the marine *Vibrio spp*. The portal of entry for this type 3 NF is a puncture wound caused by fish or marine insects and is rarely observed in Europe [11]. Type 4 describes fungal cases of candida NF, which are very rare [2, 15].

The diagnosis of NF should be considered in patients with clinical symptoms as mentioned above, but can be very difficult. To clarify the diagnosis, Wong et all described the “Laboratory Risk Indicator for Necrotizing Fasciitis” (LRINEC) score, which is based on routinely performed laboratory tests [6] (Table 1). They found a score ≥6 had a positive predictive value of 92 % and a negative predictive value of 96 %. However, this test has not been validated in larger, prospective studies. Therefore, surgical exploration remains the gold standard to definitively establish the diagnosis of necrotizing fasciitis [8, 16]. Aggressive surgical debridement (<24 h) is associated with a lower mortality [17, 18].

**Table 1:** The Laboratory Risk Indicator for Necrotizing Fasciitis (LRINEC score)

		Score
C-reactive protein (mg/l)	<150	0
	≥150	4
Leucocyte count (10^9^/l)	<15	0
	15–25	1
	>25	2
Haemoglobine (mmol/l)	>8.4	0
	6.8–8.4	1
	<6.8	2
Sodium (mmol/l)	≥135	0
	<135	2
Creatinine (μmol/l)	≤141	0
	>141	2
Glucose (mmol/l)	≤10	0
	>10	1
Total		13

Appropriate treatment of a patient with NF can only be achieved through close cooperation between the surgeon, intensivist and microbiologist.

The aim of our study was to analyse all available data of a large cohort of patients presenting with NF in four teaching hospitals in the Netherlands. Also, we determined the causative pathogens in our population, described clinical management and clinical outcome in this Dutch cohort and compared that with previous other studies.

## Methods

### Study design

The study was designed as a retrospective cohort study.

### Patients

All consecutive adult patients who were diagnosed with NF were eligible for inclusion at the Radboud University Medical Center Nijmegen (Radboudumc) (a 900 beds university hospital), the Gelderse Vallei Hospital Ede (GVH) (a 500 beds hospital), Rijnstate Hospital Arnhem (RH) (a 950 beds hospital) and Slingeland Hospital Doetinchem (SH) (a 340 beds hospital) between January 2003 and December 2013. These hospitals are located in the Central-Eastern part of the Netherlands, belonging to one surgical training region.

For inclusion, patients had to stay at the intensive care unit for at least one day. Patients were found by hospital data system, diagnostic codes and microbiological results.

### Data collection

Diagnosis of necrotizing fasciitis was proven by histopathologic examination of tissue samples or surgical findings when no tissue sample was analyzed. This means the presence of an affected fascia, which was documented in the procedure note as a necrotizing fasciitis, was diagnostic. Vital parameters (e.g., temperature, blood pressure, heart rate), clinical symptoms of the affected body part and laboratory results at presentation, as well as all demographic data were collected from the patient charts. Results of blood and wound cultures, the number of surgical interventions, operative findings, length of stay at the intensive care unit (ICU), total duration of hospitalization and the mortality rate were documented. For all patients the LRINEC score was calculated from the laboratory findings.

We considered Type 2 FN as caused by a monoculture of hemolytic streptococcus group A (*Streptococcus pyogenes*), or in rare cases caused by *Staphylococcus aureus* or hemolytic streptococcus group C or G.

And in contrast Type 1 FN was seen as caused by different combinations of anaerobic bacteria, aerobic gram negative rods from the Enterobacteriaceae group and streptococci other than *Streptococcus pyogenes*.

## Results

### Initial assessment

A total of 58 patients were included (19 Radboudumc; 15 GVH, 16 RH, 8 SH). Thirty-four patients were male (58.6 %) and 24 were female (41.4 %). The median age was 62 years (range 21–81 years).

Localisation of the fasciitis was in the central part of the body in 28 patients (48.3 %) and in one of the extremities in 21 patients (36.2 %). In 8 patients (13.8 %) there was a combination of central part of the body with one of the extremities. In one patient the head was the affected.

The most common comorbidity was cardiovascular diseases (39.7 %). Other co-morbidities included were obesity (25.9 %), diabetes mellitus (24.1 %) and malignancy (19.0 %). Thirteen patients (22.4 %) had no comorbidities.

Etiology of the necrotizing fasciitis was a minimal trauma in 16 patients (27.6 %) (median 4 days, range 1–30). Fourteen patients had undergone an operation a few days before they developed necrotizing fasciitis (median 3.3 days, range 1–60). Seven of them had undergone a sterile operation (e.g., inguinal hernia repair or lumpectomie), the other seven patients a contaminated operation (e.g., appendicitis or bowel resection) (Table 2). In 28 patients (48.3 %) there was no portal of entry or no known cause for the NF.

**Table 2:** Operations prior to necrotizing fasciitis

Sterile	Number	Contaminated	Number
Inguinal hernia repair	2	Bowel resection	4
Renal transplantation	1	Hemorrhoidectomy	2
Laparoscopic cholecystectomy	1	Appendectomy	1
Adnex extirpation	1		
Lumpectomie	1		
Vasectomy	1		
Total	7		7

**Fig. 1:**
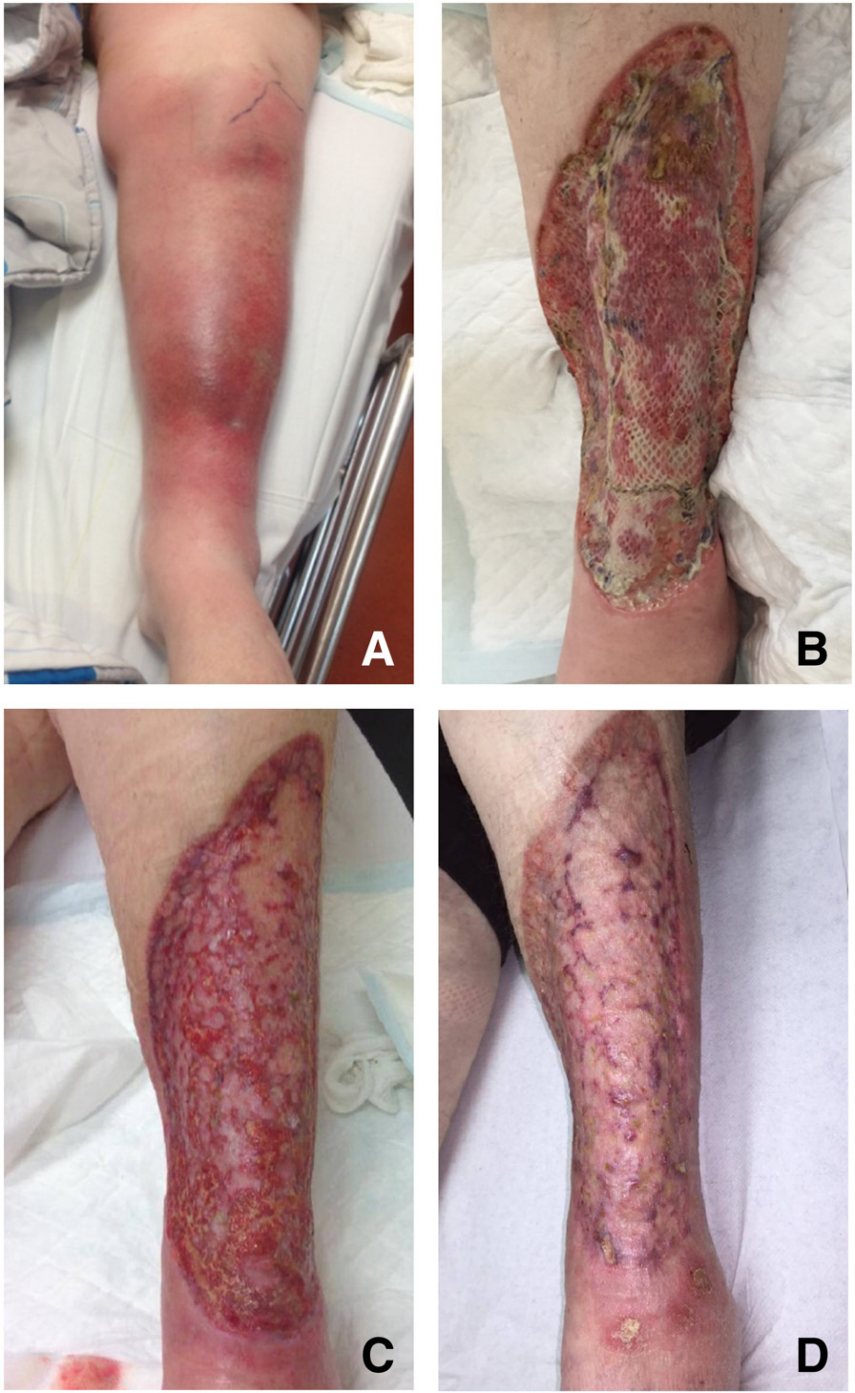
A 65 year old women with a history of diabetes mellitus, renal insufficiency and corticosteroid use, presented on the emergency room with fever, progressive pain, erythema and swelling in her left leg (**a**). Surgical debridement showed typical signs of necrotizing fasciitis. Two weeks after presentation and 10 days after VAC therapy, she has got a split skin graft (**b**). Follow up after 3 months (**c**) and 5 months (**d**) showed a well-healed wound

Nineteen patients (32.8 %) had a fever at time of presentation with a body temperature of >38.5 °C. Signs and symptoms at admission were swelling (54 cases, 93.1 %), erythema (52 cases, 89.7 %), tachycardia (33 cases, 57.9 %) and blisters (14 cases, 24.1 %). Other symptoms like crepitation or loss of sensibility were rare. Figure 1a shows a swelling and erythema matching a necrotizing fasciitis.

Forty-six patients had a LRINEC score ≥ 6 (79.3 %), 33 patients had a score ≥ 8 (56.9 %) and 8 patients had a score ≥ 10 (13.8 %). The mean LRINEC score was 7.4.

### Microbiology

All 58 patients had positive cultures. We found a type 1 necrotizing fasciitis in 35 patients (60.3 %) and a type 2 in 23 patients (39.7 %). In 9 out of 35 patients (25.7 %) with a type 1 NF, we isolated a monoculture, mostly *E. coli*. In the other 26 patients (74.3 %), a total of 61 (mixed) pathogens were isolated (Table 3).

**Table 3:** Cultures in necrotizing fasciitis type I, *n* = 35 patients

Micro organism	Monoculture	Present in mixed culture	Total
Escherichia coli	6	13	19
Klebsiella pneumoniae	1		1
Proteus mirabilis		3	3
Citrobacter freundii		1	1
Enterobacter cloacae		2	2
Serratia marcescens		1	1
Pseudomonas aeruginosa	1	1	2
Acinetobacter baumannii		1	1
Stenotrophomonas maltophilia		1	1
Aeromonas sobria		1	1
Bacillus species		1	1
Haemolytic streptococci, not group A		3	3
Enterococcus species		4	4
Streptococcus pneumoniae		1	1
Viridans streptococci		3	3
S.milleri group		6	6
Clostridium perfringens		2	2
Anaerobe gram negative rods, mainly B.fragilis	1	5	6
Anaerobic mixed culture		5	5
Mixed culture		7	7
Total cultures	9 (13 %)	61 (87 %)	70 (100 %)
Total patients	9 (26 %)	26 (74 %)	35 (100 %)

### Treatment and follow up

All patients underwent one or more operations. Thirty-nine patients (67.2 %) were operated within 24 h and 16 patients (27.6 %) underwent their first operation after 24 h. In 3 patients the exact time of operation was not clear.

Forty-nine patients (84.5 %) underwent radical necrotectomy. In 6 cases (10.3 %) an amputation was necessary. In 3 patients the NF was so extensive, that because of poor prognosis, radical necrotectomy was not conducted.

The mean number of debridement procedures of the patients who survived was 2.8 (range 1–8). In 26 patients (44.8 %) we used vacuum assisted closure therapy, which in 12 patients was followed by definitive reconstruction by split skin grafts (Fig. 1b-d). Other patients received split skin grafts without VAC® (Vacuüm Assisted Closure) therapy, primary wound closure or reconstruction by the plastic surgeon.

The mortality was 17 out of 58 patients (29.3 %). The median age of the patients who died was 64 years (range 38–72 years). The median age of the survivors was 59 years (range 21–81 years). Nine patients died within 2 days because of multi organ failure or cessation of treatment due to poor prognosis. Two patients died in the first week after admission. Six patients died several weeks or months (range 15–73 days) after admission because of a new septic period, cardiac failure or general weakness.

From 38 patients histological examinations were taken. In two cases an autopsy was performed. All patients showed typical signs of NF.

The mean duration of hospitalization of the patients who survived was 46 days (range 11–166). The mean stay on the intensive care unit was 11 days (range 1–42).

## Discussion

Necrotizing fasciitis is a part of the Necrotizing Soft Tissue Infections (NSTI) and is a rare life threatening disease that still has a high mortality and morbidity. We included 58 patients with necrotizing fasciitis in four different hospitals. To our knowledge, this study is the first Dutch study and involves one of the largest European cohorts. Previous large studies have been conducted in Asia and Australia [5–7, 19, 20]. Because we exclusively wanted to include patients with the fulminant form of necrotizing fasciitis, patients with NF admitted for at least one day in the intensive care unit, were included. Patients with a subacute fasciitis [21] or a doubtful diagnosis were excluded from our study.

Many patients presented with classical symptoms like erythema, swelling and tachycardia, had abnormal blood results and had a clear minimal trauma or a surgical wound as the portal of entry. Despite the classical presentation, up to 30 % was not operated on within 24 h after admission. This is consistent with the literature, which shows even higher numbers of delay of surgical treatment up to 40 % [7, 20]. Also, Goh et all describes 71.4 % misdiagnosis of NF as cellulitis or abscess in their systematic review [12]. This illustrates the diagnostic dilemma that is present in a large number of patients.

The LRINEC score can be a useful tool to help diagnose NF [6]. In the initial study, a score of 6 or above was shown to have a positive predictive value of 92 % and a negative predictive value of 96 % [6]. However, no prognostic studies for validating this score, and the cut-off value of ≥6, are available [22]. Twenty-one percent of our patients had a LRINEC score below 6. However, this was a selected group in which the diagnostic process had already been completed. The LRINEC score was used retrospectively and therefore did not aid in the diagnosis. If we want to use the LRINEC score in this way, an adequate validation is necessary.

The most common risk factor for NF in the literature is diabetes mellitus [5, 7, 11, 12, 23, 24], however, there are major differences between studies (Bucca at all 21 %, Wong at all 70.8 %) [6, 25]. In our study only 24.1 % of the patients had diabetes. The most common comorbidity we found was cardiovascular diseases in almost 40 % of the patients. Over 50 % of them had a serious cardiac event like infarction or arrhythmia. The other patients only had hypertension. The number of patients with no comorbidities (22.4 %) is comparable to other studies [5, 19].

Many of the larger studies on NF are published in South East Asia or Australia. These countries live in close relation to marine life. In addition to type 1 and type 2 NF, type 3 was described regularly. We had no patients in our study with NF type 3. Huang described 11.9 % wound cultures with *Vibrio spp*, it was the most common pathogen leading to bacteraemia (29.5 %) in their population [7]. This difference in pathogens may also have influenced the course of the disease and outcome of patients with NF. In our study we found 60.3 % of patients with type 1 NF and 39.7 % with type 2 NF. This is similar to other studies [3, 8, 9]. Studies with higher numbers of type 2 NF describe up to 63.3 % type 2. These studies used other criteria to distinguish between type 1 and 2, classifying all monomicrobial infections as type 2 [7, 24, 26].

The treatment of patients with NF is challenging and consists of adequate surgical debridement, supportive care by the intensivist and starting broad spectrum antimicrobials [25, 27]. The choice of antibiotics depends on the suspected causative microorganism(s), part of the body that is affected and clinical picture. Antibiotic therapy can be narrowed down as culture results are known. The most important factor is early surgical aggressive debridement, which is associated with a lower mortality when performed within 24 h [17, 18].

Mortality in our study population was 29 %, which is slightly higher than in other reports of patients with NF [5, 7, 10, 19, 20, 24, 25, 28]. This can be explained by the different inclusion criteria and possible different microorganism(s). Due to the inclusion criteria of at least 1 day ICU-stay we possibly included a more seriously ill population compared to other reports.

Recent years have shown an increase in the use of the vacuum assisted therapy [29]. We used the VAC® therapy in nearly half of our patients with good results.

Our study was limited by the retrospective character. Another limitation is, because of the rarity of NF, patients of four different hospitals are included. Different routines in the hospitals, although they belong to the same training regions, and the relative long period of inclusion can explain some missing data.

## Conclusions

We present the first Dutch and the largest European study of patients with necrotizing fasciitis. Our study reaffirmed that NF is a life threatening disease with a high mortality. Early diagnosis and adequate treatment are necessary to improve the clinical outcome. Clinical awareness of necrotizing fasciitis remains pivotal.

Our recommendations for further research are a prospective study to validate the LRINEC score and to explore the correlation between the score and clinical outcome.

## Abbreviations

GVH, Gelderse Vallei Hospital Ede; ICU, Intensive Care Unit; LRINEC, Laboratory Risk Indicator for Necrotizing Fasciitis; NF, Necrotizing Fasciitis (NF); NSTI, Necrotizing Soft Tissue Infections; Radboudumc, Radboud University Medical Center Nijmegen; RH, Rijnstate Hospital Arnhem; SH, Slingeland Hospital Doetinchem; VAC®, Vacuüm Assisted Closure.
